# Differential effects of the venoms of Russell’s viper and Indian cobra on human myoblasts

**DOI:** 10.1038/s41598-024-53366-9

**Published:** 2024-02-07

**Authors:** Husain Bin Haidar, José R. Almeida, Jarred Williams, Bokai Guo, Anne Bigot, Subramanian Senthilkumaran, Sakthivel Vaiyapuri, Ketan Patel

**Affiliations:** 1 School of Biological Sciences, University of Reading, Reading, RG6 6UB UK; 2 Kuwait Cancer Control Centre, Ministry of Health, Kuwait City, Kuwait; 3 School of Pharmacy, University of Reading, Reading, RG6 6UB UK; 4 INSERM, CNRS, Institute of Myology, Centre of Research in Myology, Sorbonne Universities, UPMC University Paris, Paris, France; 5 Manian Medical Centre, Erode, Tamil Nadu India

**Keywords:** Cell biology, Chemical biology

## Abstract

Local tissue damage following snakebite envenoming remains a poorly researched area. To develop better strategies to treat snakebites, it is critical to understand the mechanisms through which venom toxins induce envenomation effects including local tissue damage. Here, we demonstrate how the venoms of two medically important Indian snakes (Russell's viper and cobra) affect human skeletal muscle using a cultured human myoblast cell line. The data suggest that both venoms affect the viability of myoblasts. Russell’s viper venom reduced the total number of cells, their migration, and the area of focal adhesions. It also suppressed myogenic differentiation and induced muscle atrophy. While cobra venom decreased the viability, it did not largely affect cell migration and focal adhesions. Cobra venom affected the formation of myotubes and induced atrophy. Cobra venom-induced atrophy could not be reversed by small molecule inhibitors such as varespladib (a phospholipase A_2_ inhibitor) and prinomastat (a metalloprotease inhibitor), and soluble activin type IIb receptor (a molecule used to promote regeneration of skeletal muscle), although the antivenom (raised against the Indian ‘Big Four’ snakes) has attenuated the effects. However, all these molecules rescued the myotubes from Russell’s viper venom-induced atrophy. This study demonstrates key steps in the muscle regeneration process that are affected by both Indian Russell’s viper and cobra venoms and offers insights into the potential causes of clinical features displayed in envenomed victims. Further research is required to investigate the molecular mechanisms of venom-induced myotoxicity under in vivo settings and develop better therapies for snakebite-induced muscle damage.

## Introduction

Skeletal muscle damage around the bite site with the loss of muscle mass and function is one of the major complications associated with snakebite envenoming (SBE)^1^. This negatively impacts the quality of life for victims and is a significant contributor to SBE-induced permanent disabilities^2^. Snake venom metalloproteases (SVMPs), phospholipase A_2_ (PLA_2_), and three-finger toxins (3FTX) are the most recognised mediators of local myonecrosis found in snake venoms^3,4^. SVMPs mainly act through the degradation of the extracellular matrix (ECM) around the skeletal muscle, damaging blood vessels and leading to serious implications on muscle regeneration^5^. PLA_2_s cause the degradation of skeletal muscle fibres through their characteristic disruptions in the cell membrane^6^. Similarly, 3FTXs induce myonecrosis through pore formation on cell membranes leading to the release of intracellular contents, including biomarkers such as creatine kinase and lactate dehydrogenase^5^. Skeletal muscle has a robust ability to regenerate through the orchestration of various cellular activities following damage. The key to this regenerative process is the resident muscle stem cells called satellite cells. These mononucleated cells are found in a quiescent state in an undamaged muscle^7^. However, following damage, they undergo several changes including proliferation, migration to the site of damage, fusion and hypertrophy, all of which are required to replace the lost muscle fibres^8^. Perturbations in any of these processes result in attenuated or improper regeneration.

The current understanding of the pathogenesis of SBE-induced myonecrosis points out that the individual and/or synergistic actions of venom toxins directly or indirectly contribute to this process^9^. Notably, the differential expression of key toxins in elapid (e.g. cobra) and viper (e.g. Russell’s viper) venoms exhibit varying patterns in clinical profiles and muscle damage^2^. Elapid venoms are generally shown to possess high levels of membranolytic PLA_2_s and 3FTXs, while viper venoms are rich in SVMPs, and PLA_2_s^10^. As a result, the incidence of long-term musculoskeletal disorders in victims of elapid bites is relatively low compared to viper bites, in which the process of innate muscle regeneration is significantly impeded^2^. The underlying molecular mechanisms resulting in poor skeletal muscle regeneration following exposure to viper and some elapid venoms are poorly understood^11^. Given that muscle regeneration is underpinned by several phases, it is important to identify which step is affected by a particular venom to understand the basis of the musculoskeletal disorders exhibited by victims.

Currently, antibody (antivenom)-based therapy is the only effective method for neutralising the venom toxicity^12^. While antivenoms are critical in saving lives, they have serious limitations, such as batch variation, high production cost and importantly, low efficacy in preventing venom-induced local envenomation effects including muscle damage^13^. Several alternative methods have been studied in recent years to develop the next generation of antivenoms^14^. The repurposing of small molecule inhibitors such as PLA_2_ inhibitor, varespladib has shown the potential to revolutionise SBE treatments^15^. However, the pharmacological assessment of small molecule inhibitors to date has mainly focused on the neutralisation of venom-induced lethality and systemic effects^16^. Clinical trials are currently ongoing to determine the efficacy of one (varespladib) of these repurposed broad-spectrum therapeutics for the SBE^17^. SVMP inhibitors such as prinomastat are also being widely studied to determine their effects in neutralising venom-induced complications. However, the impact of these small molecule inhibitors and other potential molecules such as regenerative medicine approaches that safeguard muscle has not been studied in sufficient detail to determine their efficacy in neutralising venom-induced muscle damage using robust cell-based assays and in vivo animal models. From a translational perspective, the AB1190, a human-immortalised myoblast cell line, emerges as an attractive research tool for screening a range of therapeutic molecules and pathological studies relating to skeletal muscle damage^18^. They can be cultured in vitro as undifferentiated cells in growth media and then differentiated into multinucleated myotubes. This cell line acts as a robust system for dissecting the mechanisms of myogenesis and its modulation by pharmacological agents or biologically active compounds^19^. Here, we evaluated the impact of the venoms of two medically important Indian snakes, Russell’s viper (*Daboia russelii*) and cobra (*Naja naja*) on myoblasts as their bites are known to induce significant muscle damage in humans. Moreover, we determined the effects of varespladib, prinomastat and soluble activin type IIB-receptor [sActRIIB, a molecule that has been widely used to promote muscle growth under diverse pathological settings^20^ as well as a polyvalent antivenom (raised against the venoms of Russell’s viper, cobra, krait, and saw-scaled viper) to mitigate the muscle damage induced by these venoms.

## Methods

### Materials used in this study

Lyophilized venoms of *D. russelii* (lot number: 0120) and *N. naja* (lot number: 0120) were obtained from Kentucky Reptile Zoo, USA. These are pooled venoms collected from multiple specimens of the same species. The venoms were dissolved in phosphate-buffered saline (PBS) to prepare stock solutions and further diluted at required concentrations in a culture medium before use. The antivenom raised against the venoms of the Indian ‘Big Four’ snakes (Russell’s viper, cobra, krait, and saw-scaled viper) was obtained from Bharat Serums and Vaccines Limited, India (Batch Number: A05317068). Small molecule inhibitors, varespladib and prinomastat were obtained from Sigma Aldrich, UK. Recombinantly expressed soluble activin type IIB-receptor (sActRIIB) was a kind gift from Dr Ritvos, Helsinki, Finland.

### Cell culture

The AB1190 is an immortalised myoblast cell line developed by Dr Bigot at INSERM, France using muscle cells isolated from the paravertebral muscle of a disease-free 16-year-old male. This cell line was used as an in vitro tool in this study to determine the impact of venoms. AB1190 myoblasts were routinely maintained in 75 cm^2^ flasks in skeletal muscle cell growth medium (GM) with the supplied supplement mix (Product Number: C-23160, PromoCell, Germany), and by incubating in a humidified atmosphere of 5% CO_2_ at 37 °C. The medium was refreshed every other day. The enzymatic disaggregation was performed using 3 mL of recombinant trypsin, TrypLE™ (ThermoFisher Scientific, UK) for sub-culturing to avoid high confluence and contact inhibition or transformation, or for seeding new plates. All our experimental studies were performed in passages 6–12 of AB1190 cells. No significant changes in any parameters including doubling times were noticed during these passages.

### Cell viability assay

The CellTiter96® AQ_ueous_ one solution Cell Proliferation Assay kit that includes 3-(4,5-dimethylthiazol-2-yl)-5-(3-carboxymethoxyphenyl)-2-(4-sulfophenyl)-2H-tetrazolium (MTS) (Promega, UK) was used in AB1190 cells to determine the effects of venoms on myoblast viability. The cells were seeded at a density of 10,000 cells (the use of more than 10,000 cells results in high confluence and random myotube formation at 24 h, hence, we used only 10,000 cells in this assay) per well of a 96-well plate in 100 µL of growth medium overnight at 37 °C and 5% CO_2_ for the cells to adhere. These undifferentiated myoblasts were then exposed to different venom concentrations dissolved in GM for 24 h. A control group was added with only fresh GM. All experiments were performed in triplicates. Following incubation with venoms, 10 µL of MTS reagent was added to each well. The 96-well plate was then incubated for two hours at 37 °C and 5% CO_2_. Cell viability was estimated based on the absorbance of the formazan (developed from MTS reagent) product, which was measured at 490 nm using a spectrofluorometer (SpectraMax® ID3, Molecular Devices, UK). In addition, the cell morphology was observed and imaged using a digital cell imager through a 10X objective (EVOS XL, ThermoFisher Scientific, UK).

### Cell migration assay

To examine the effect of venoms on cell migration, an in vitro cell-tracking assay was used. Human myoblasts were seeded at a low confluence (3000 cells per well in a 12-well plate) with 1 mL of GM and incubated overnight at 37 °C with 5% CO_2_. Before time-lapse imaging, the microscope’s environmental chamber was equilibrated to 37 °C and 5% CO_2_. The cells were incubated with different concentrations (1.25, 2.5 and 5 µg/mL) of venoms in GM and imaged for 24 h using a brightfield TIE microscope (Nikon, UK) at a rate of one frame per 10 min with a 10X objective. The selection of non-toxic concentrations of venoms to use in this assay was performed based on the data obtained from the MTS assay. For the negative control, only GM (without venom) was used. For the quantitative analysis of the cell migration, two parameters were compared: relative accumulated distance and speed. Individual cells were tracked on Image J using the MTrackJ plugin. Cells for tracking were selected based on their isolated position at the beginning of the assay. Multiple cells were tracked within a single field of view and several (minimum five) views were recorded for each well. A minimum of three wells were assessed for each concentration of venoms. The cell position was tracked every ten minutes for a total duration of 24 h. The software then tracked the cumulative path permitting the calculation of distance travelled as well as average speed. Results are given as percentage changes (for easier understanding) compared to the control which was taken as 100%.

For a scratch assay, 40,000 cells were seeded into each well of a 24-well plate and allowed to adhere overnight at 37 °C at 5% CO_2_. A scratch was then made using a 10 µL pipette tip across the face of the well and the media was removed. The cells were then washed once with PBS and fresh media containing various concentrations of venoms were added. The cells were then placed into a prewarmed microscope chamber at 37 °C and 5% CO_2_ and imaged every 10 min for 24 h with a 10 × objective using a TIE inverted microscope (Nikon, UK). Individual cells were tracked on Image J and the time taken for the first cell to cross the scratch and contact one another was recorded.

### Focal adhesion staining

Myoblasts were fixed with 4% (w/v) paraformaldehyde (PFA) for 20 min and then washed twice in PBS (one minute each). Cell membranes were partially disrupted using a permeabilisation buffer [20 mM HEPES pH 7, 300 mM sucrose, 50 mM NaCl, 3 mM MgCl_2_ and 0.5% (v/v) Triton X-100] for 15 min, followed by three washes with PBS. The non-specific binding sites were blocked by incubating the cells in a blocking buffer [10% (v/v) goat serum in PBS] for 30 min at room temperature. The cells were then incubated with the primary anti-paxillin antibody (Y113) (1:200 dilution; Abcam, UK) for staining focal adhesions overnight at 4 °C and washed with PBS five times (three minutes each). Cells were then incubated with appropriate secondary antibodies for one hour in the dark at room temperature. To stain actin, rhodamine-conjugated phalloidin (1:50 dilution, ThermoFisher Scientific, UK) was used. Images were taken using a Nikon A1R Confocal microscope through a 100X objective. Image analysis was performed using ImageJ software. Focal adhesions were assessed to be present when the expression of paxillin co-localised to the phalloidin domain. Stressed fibres were assessed to be present when they met critical size parameters as reported previously^21^.

### Cell differentiation assay

Myoblast fusion analysis was conducted to evaluate the effect of venoms on myogenic differentiation. Firstly, the coverslips were prepared by sterilising with 100% ethanol for 15 min followed by acid etching treatment (2.5 M HCl) for 30 min. The coverslips were then washed with distilled water and exposed to UV light for two hours on each side. Etched coverslips were placed on to 12-well plates before seeding 45,000 cells per well. Myoblasts in 100 µL of GM were incubated at 37 °C at 5% CO_2_ and allowed to culture until they reached a minimum of 85% confluence, with media changes performed every other day. Then, the GM was replaced with differentiation media (DM) composed of high glucose DMEM (Gibco, UK) supplemented with 1% (v/v) penicillin–streptomycin (Gibco, UK) and 5% (v/v) horse serum (Gibco, UK), with the addition of different concentrations of venoms (1.25, 2.5 and 5.0 μg/mL). The myoblasts were allowed to differentiate for three days at 37 °C and 5% CO_2_. After this period, the control cells (without venom) fused into myotubes. The characteristic elongated morphology of myotubes was identified using microscopic analysis. Lastly, the myotubes were fixed using 2% (w/v) PFA in PBS followed by immunostaining (using 1:200 pan Myosin heavy chain antibody (A4.1025) made in-house from the A4.1025 hybridoma cell line^22^) and imaging. Fusion was assessed to have taken place when a minimum of three nuclei were in an MHC-positive cell. The percentage of fusion index (FI) was used as a parameter to quantify and analyse the effects of venom treatments on myoblast fusion. FI was calculated as the percentage of total nuclei incorporated in myotubes in line with total nuclei found in a field of view as determined by immunofluorescence microscopy images. The number of nuclei and myotubes were calculated manually by enlarging the images.

Similarly, myotubes fixed in 2% (w/v) PFA from fusion and atrophy assays were washed three times in PBS for 5 min each followed by permeabilisation, washing again with PBS, and blocking with 5% (v/v) FBS for 30 min at room temperature. Human myotubes were then incubated with pan Myosin antibodies (1:200) overnight at 4 °C. Cells were then washed three times for 10 min each with PBS and then the secondary antibody, Alexa Fluor 488-conjugated goat anti-mouse antibody (1:200, ThermoFisher Scientific, UK)] was added to cells and incubated for 1 h at room temperature in the dark. The nuclei of terminally differentiated muscle cells were stained with a mounting medium (Dako, UK) containing 4’, 6-diamidino-2-phenylindole (DAPI) (2.5 µg/mL). Fluorescence images were captured using an epifluorescence microscope with a 5X objective (Zeiss AxioImager, UK). All image analysis was performed in ImageJ.

### Muscle atrophy assay

For the investigation of venom-induced muscle atrophy, 45,000 cells were seeded per well and cultured onto 16 mm diameter coverslips in 12-well plates. After reaching at least 85% confluence, terminal differentiation was induced by culturing cells in DM for three days at 37 °C and 5% CO_2_, as described above, but without venoms. On day three of culture in DM, the myotubes were fully differentiated. Subsequently, the DM was replaced with DM containing different concentrations (1.25, 2.5 and 5.0 μg/mL) of venoms or fresh DM for controls. The cells were then cultured for an additional 24 h. In the end, the resulting myotubes were fixed in 2% (w/v) PFA and stored in PBS for further analysis. The mean area of myotubes was used for assessing myotube atrophy using ImageJ.

The atrophy assay was also used to assess the effects of small molecule inhibitors such as varespladib and prinomastat, polyvalent antivenom and sActRIIB (a gift from Dr Ritvos, Helsinki, Finland). After three days of differentiation, resulting myotubes were divided into different groups: (1) negative controls, in which terminally differentiated cells were incubated in medium alone, (2) venom-treated group, in which myotubes were exposed to *D. russelii* or *N. naja* venom and (3) venom and inhibitors, in which cells were exposed to media with venom and potential treatments (inhibitors, antivenom or sActRIIB). The venom and inhibitors were preincubated for five minutes before adding to the cells. The treatments with inhibitors lasted for one day. The area of at least 60 myotubes was measured for each condition to analyse the impact of venoms on atrophy.

### Statistical analysis

All statistical analysis was performed using GraphPad Prism. The values are presented as mean ± standard error of the mean (SEM). One-way analysis of variance (ANOVA) was used to calculate the significant difference between groups, followed by the Dunnett post hoc test (comparisons against control) or a Bonferroni post hoc test.

## Results

### *D. russelii* and *N. naja* venoms affect the viability of human myoblasts

**Figure 1:**
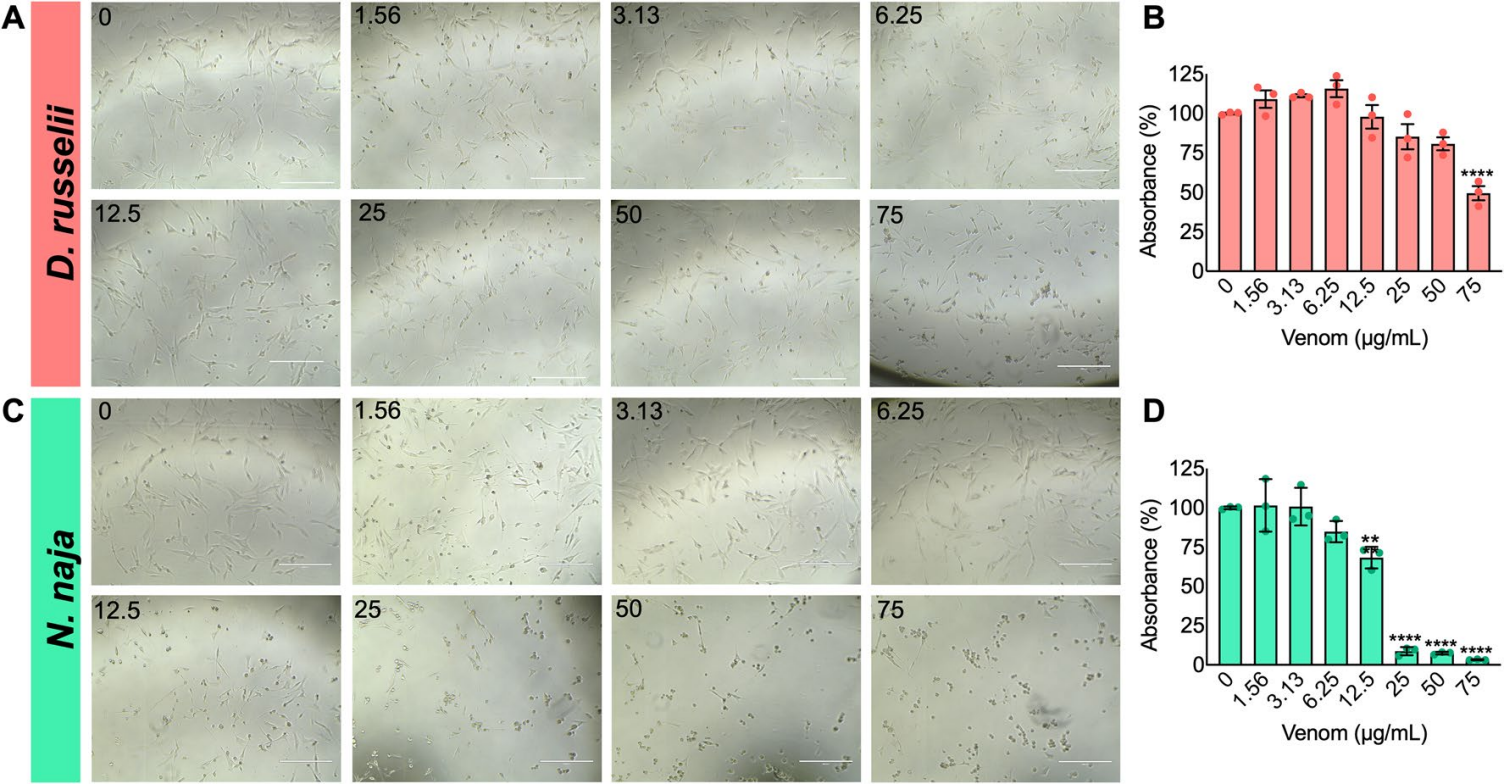
Effect of *D. russelii* and *N. naja* venoms on the viability of undifferentiated AB1190 myoblasts. Representative phase contrast images of human myoblasts incubated with different concentrations of *D. russelii* (**A**) and *N. naja* (**C**) venoms are shown. Cell viability was quantified using an MTS assay in the presence of *D. russelii* (**B**) or *N. naja* (**D**) venom and represented as the percentages in comparison to the controls. Data represent mean ± SEM (n = 3). *P* values (**p < 0.01 and ****p < 0.0001) shown were as calculated by one-way ANOVA followed by Dunnett’s post hoc test using GraphPad Prism. The scale bars represent 400 µm.

To evaluate the impacts of *D. russelii* and *N. naja* venoms on muscle, we performed a range of experiments using the human AB1190 myoblast cell line. A cell viability assay was performed using the MTS tetrazolium compound in the presence and absence of venoms. Varying concentrations of whole venoms were added to undifferentiated myoblasts and incubated for 24 h before analysing the cell viability. The results demonstrate that both *D. russelii* and *N. naja* venoms caused a significant decrease in cell viability (Fig. 1). *D. russelii* venom only affected the cell viability at a high concentration (75 µg/mL) (Fig. 1A,B). However, *N. naja* venom significantly decreased cell viability at concentrations of 12.5 µg/mL and above (Fig. 1C,D). These results were corroborated by microscopic analysis, which demonstrated phenotypic changes in myoblast cells visually at higher concentrations of venoms (Fig. 1A,C). These data suggest that both venoms affect the viability of human myoblasts but at varying concentrations.

### Migration of human myoblasts was affected by *D. russelii* and *N. naja* venoms

**Figure 2:**
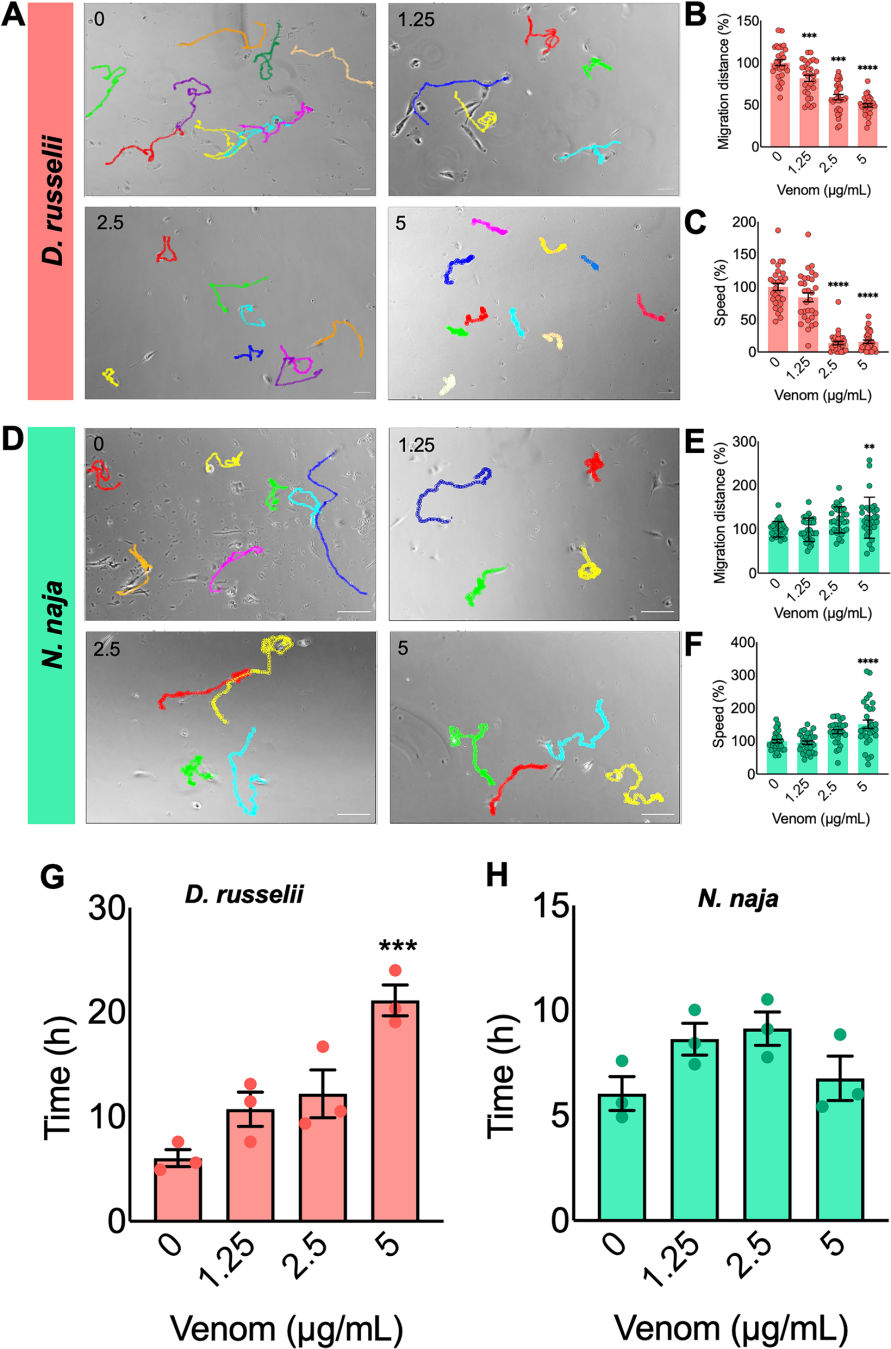
Effect of venoms on the migration of human myoblasts. Representative images of migration trajectories of AB1190 myoblasts are shown for the controls along with *D. russelii* (**A**) or *N. naja* (**D**) venom group after exposure to 1.25, 2.5 and 5 µg/mL of venoms for 24 h. By tracking selected individual cells, migration distance and speed were calculated using ImageJ with the MtrackJ plugin extension. To allow easier comparison, the mean values of the controls were taken as 100% to calculate the values for venom-treated samples. The bar graphs represent the relative cell migration distance (**B**,**E**) and migration speed (**C**,**F**) of cells. Data represent mean ± SEM (30 cells were tracked for each venom concentration). *P* values (**p < 0.01, ***p < 0.001, and ****p < 0.0001) shown were as calculated by one-way ANOVA followed by Dunnett’s post hoc test using GraphPad Prism. The scale bars represent 100 µm. Similarly, the impact of the same concentrations of *D. russelii* (**G**) and *N. naja* (**H**) venoms was analysed using a scratch assay in myoblasts for 24 h. The graphs represent the time taken for cells to traverse the scratch made in the myoblast monolayer and make contact on the other side. The time taken for the cells from one side to contact the cells on the other side was compared with the controls. Data represent mean ± SEM (n = 3). *P* value (***p < 0.001) shown was calculated by one-way ANOVA followed by the Bonferroni Post hoc test using GraphPad Prism.

Successful muscle development and regeneration rely on the migration of myoblasts^23^. Therefore, the migratory capacity of AB1190 cells in the absence and presence of various venom concentrations was analysed. Figure 2A,D show the migration trajectories of selected cells (as shown in coloured lines for individual cells) after 24 h of incubation with venoms. Two parameters of cell migration (i.e., distance travelled in 24 h and relative speed in comparison to the controls) were assessed for selected cells. The results demonstrate that all concentrations of *D. russelii* venom significantly decreased the migration distance (Fig. 2B) although the migration speed was reduced only at 2.5 and 5 µg/mL (Fig. 2C). Notably, the migration was not affected at the lower concentrations of *N. naja* venom (1.25 and 2.5 µg/mL). However, the migration distance (Fig. 2E) and speed (Fig. 2F) were both slightly increased at the high concentration of 5 µg/mL. To corroborate these results, a scratch assay was performed to assess the migratory capacity of myoblasts exposed to various concentrations of venoms. Following the scratch, the ability of myoblasts to migrate was significantly decreased by *D. russelii* venom at 5 µg/mL, as shown through a significant increase in the time taken for cells from both sides of the scratch to make contact (Fig. 2G). However, there was no significant effect observed by any of the concentrations of *N. naja* venom. These data suggest that the venom of *D. russelii* affects the migration of myoblasts, but *N. naja* venom does not have any major impacts on cell migration.

### Focal adhesions were affected by *D. russelii* venom

**Figure 3:**
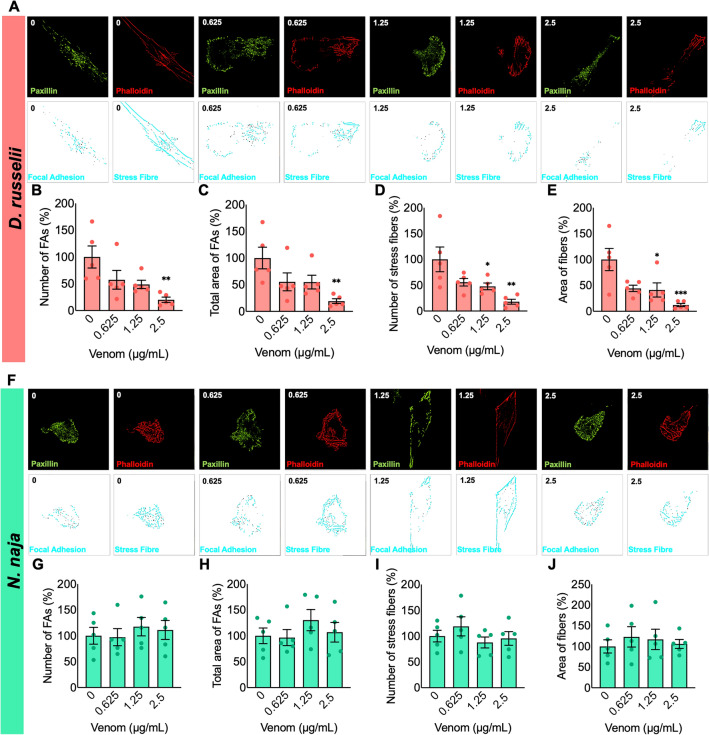
Effect of venoms on focal adhesions and cytoskeletal organisation of AB1190 myoblasts. Representative confocal images of cells incubated with different concentrations of *D. russelii* (**A**) or *N. naja* (**F**) venom and stained with anti-paxillin antibodies and rhodamine-conjugated phalloidin are shown. The processed images showing focal adhesion and stress fibres are also shown below the fluorescent images. Quantitative analysis of the number, and area of focal adhesions (FAs) and stress fibres after treatment with *D. russelii* (**B**–**E**) or *N. naja* (**G**–**J**) venom for 24 h are shown. Data represent mean ± SEM (n = 5). *P* values (*p < 0.05, **p < 0.01 and ***p < 0.001) shown were as calculated by one-way ANOVA followed by Dunnett’s post hoc test using GraphPad Prism. Images were taken at a magnification of 100X.

Focal adhesions and stress fibres form complex protein–protein interactions that produce, sense, and transmit mechanical tensions that are required for cell migration^24^. To ascertain the influence of various concentrations of venoms on cell migration, the focal adhesions and filamentous actin (F-actin or stress fibres) were analysed based on the co-localisation of paxillin and phalloidin^21^. The results indicate that paxillin and phalloidin levels were reduced by *D. russelii* venom (Fig. 3A). Myoblasts exposed to 2.5 µg/mL of *D. russelii* venom exhibited reduced focal adhesions, their area and the number of stress fibres compared to the controls (Fig. 3B–E). However, *N. naja* venom did not affect the number, average area, or total area of focal adhesions or F-actin filaments in any of the concentrations tested (Fig. 3F–J). These data suggest that the *D. russelii* venom significantly affects the focal adhesions and actin filaments while cobra venom does not have an impact on them.

### Both venoms affected the fusion of myoblasts and induced atrophy

**Figure 4:**
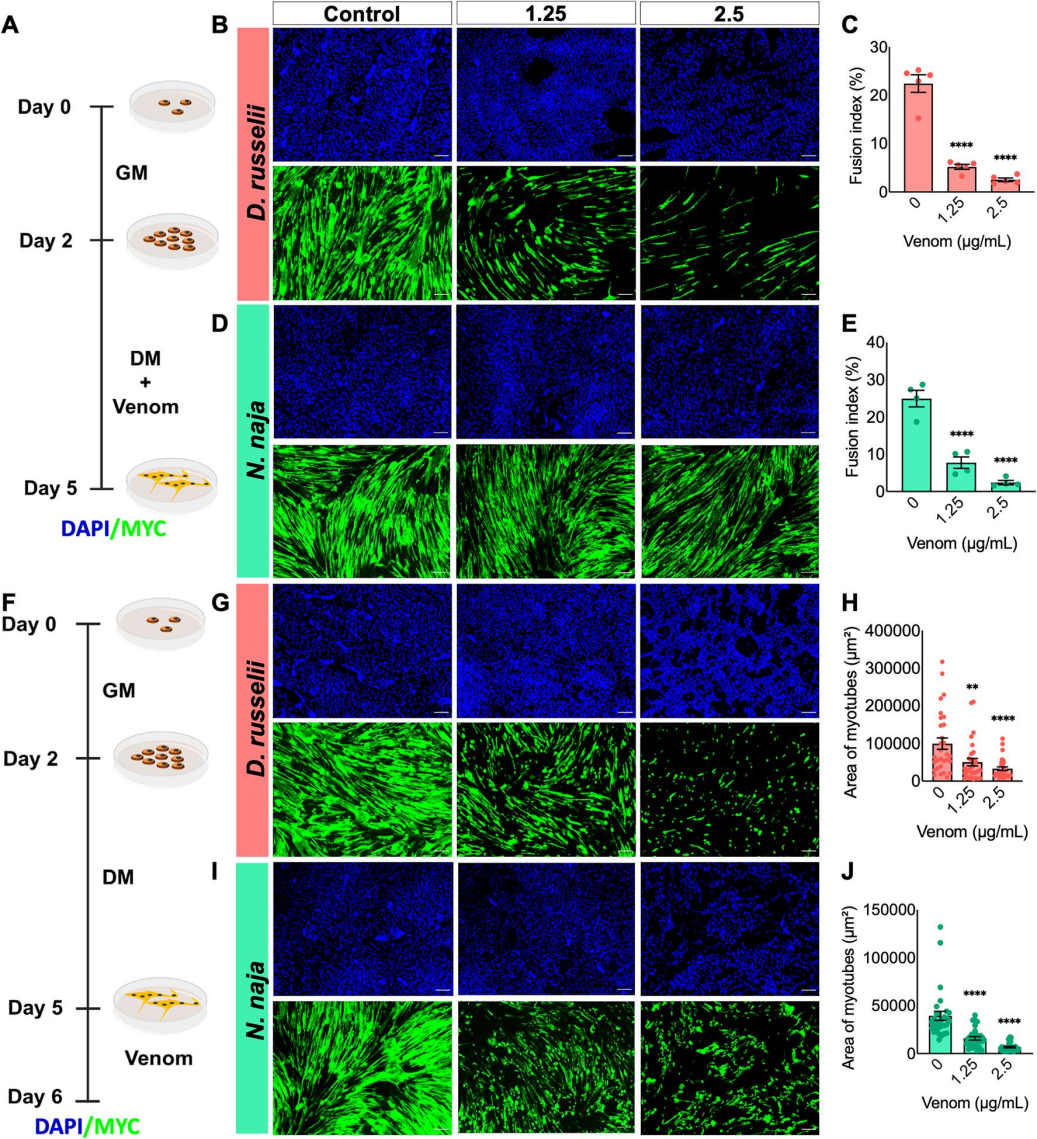
Impact of venoms on muscle fusion and atrophy. (**A**) Schematic representation of the experimental procedure used to investigate the effect of venoms on myoblast fusion. Representative images of cultured cells incubated with various concentrations of *D. russelii* (**B**) and *N. naja* (**D**) venoms are shown. Their percentage of fusion index (FI) (**C**,**E**) was quantified in comparison to the controls. (**F**) Schematic illustration of the atrophy assay. Terminally differentiated myotubes were exposed to different concentrations of *D. russelii* (**G**) and *N. naja* (**I**) venoms. The area of myotubes calculated for each venom is provided in **H** and** J**. Negative controls (0) are cells incubated with only differentiation medium and they are included for comparison in all experiments. Data represent mean ± SEM (30 myotubes were assessed for each treatment). *P* values (**p < 0.01 and ****p < 0.0001) shown were as calculated by one-way ANOVA followed by Dunnett’s post hoc test using GraphPad Prism. The scale bars represent 100 µm.

**Figure 5:**
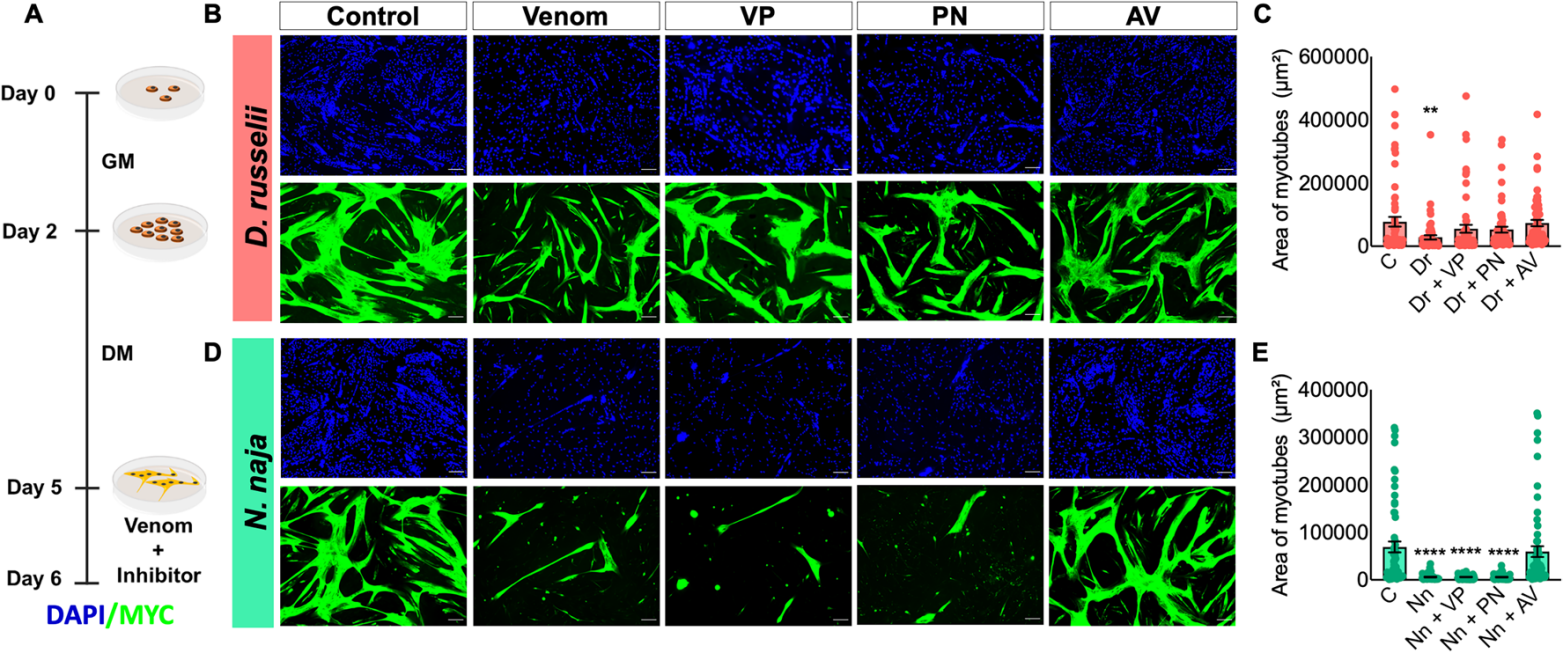
The ability of varespladib, prinomastat and anti-venom to attenuate venom-induced muscle atrophy. (**A**) Schematic illustration of the experimental procedure used to assess the impact of  different inhibitors on atrophy caused by venoms. Representative images (**B**) and quantification of the area of myotubes (**C**) that were incubated with 1.25 µg/mL *D. russelii* venom (Dr) in the absence or presence of 10 µM varespladib (VP), 10 µM prinomastat (PN) or 100 µg/mL antivenom (AV) are shown. The same procedure and quantification were repeated for 1.25 µg/mL *N. naja* venom (**D**,**E**). Negative controls (**C**) are represented by myotubes incubated with only a differentiation medium and they were included for comparison. Data represent mean ± SEM (60 myotubes were assessed for each treatment). *P* values (**p < 0.01 and ****p < 0.0001) shown were as calculated by one-way ANOVA followed by Dunnett’s post hoc test using GraphPad Prism by comparing all treated groups with the negative controls (**C**). The scale bars represent 100 µm.

To determine the impact of venoms on myogenic differentiation, the undifferentiated myoblasts were cultured until they reached 85% confluency and then, the differentiation was induced by replacing the GM with DM containing various concentrations of venoms for three days (Fig. 4A). To estimate the fusion of myoblasts and monitor this process, nuclei were visualised using DAPI (a blue-fluorescent DNA stain) and myotubes were stained using a terminal differentiation marker, MYC (myosin heavy chain, green). After three days of differentiation, the formation of typical myotubes was observed in the controls. However, *D. russelii* venom caused a significant reduction in the number of nuclei (as demonstrated through the FI) found in MHC-expressing cells (myotubes) in both concentrations of venom used (Fig. 5B,C). The FI was reduced from around 25 to 5% in venom-treated samples. Similarly, *N. naja* venom impaired the development of human myotubes (Fig. 5D,E) as demonstrated by a pronounced decrease in the FI (from around 30% to less than 10%) and the proportion of nuclei inside mature myotubes.

To determine the effects of venoms on later stages of muscle development, the myotubes were treated with various concentrations of venoms using a protocol like that described above. However, in this assay, the venoms were added to the medium only after the complete formation of myotubes (i.e., 3 days after inducing differentiation) (Fig. 4F). Both *D. russelii* (Fig. 4G,H) and *N. naja* (Fig. 4I,J) venoms caused a significant decrease in the average area of myotubes at both concentrations used. These data highlight that both venoms impair myoblast fusion and induce muscle atrophy.

### Varespladib and prinomastat prevent *D. russelii* venom-induced atrophy

To determine if varespladib and prinomastat can prevent venom-induced muscle atrophy, they were tested in atrophy assay in comparison to currently used antivenom in clinical practice for *D. russelii* and *N. naja*. The experimental timeline/procedure used here is highlighted in Fig. 5A. The control group exhibited large fibres expressing high levels of MYC. The myotubes treated with venoms (1.25 µg/mL) showed a significant decrease in the area of myotubes, reflecting the atrophy induced by these venoms (Fig. 5B–E). The polyvalent antivenom (100 µg/mL) is effective in preventing the decrease in the myotube area caused by both venoms. Myotubes treated with *D. russelii* venom pre-incubated with varespladib and prinomastat (10 µM) were the same size as control myotubes indicating that these inhibitors reversed the actions of *D. russelii* venom. However, these inhibitors showed no effects on *N. naja* venom-treated myotubes (Fig. 5D,E). These data suggest that the antivenom is effective in preventing atrophy induced by either venom, but the inhibitors (varespladib and prinomastat) are only effective for *D. russelii* not for *N. naja* venom. Notably, the inhibitors alone did not result in any adverse effects on myotubes (Figure S1).

**Figure 6:**
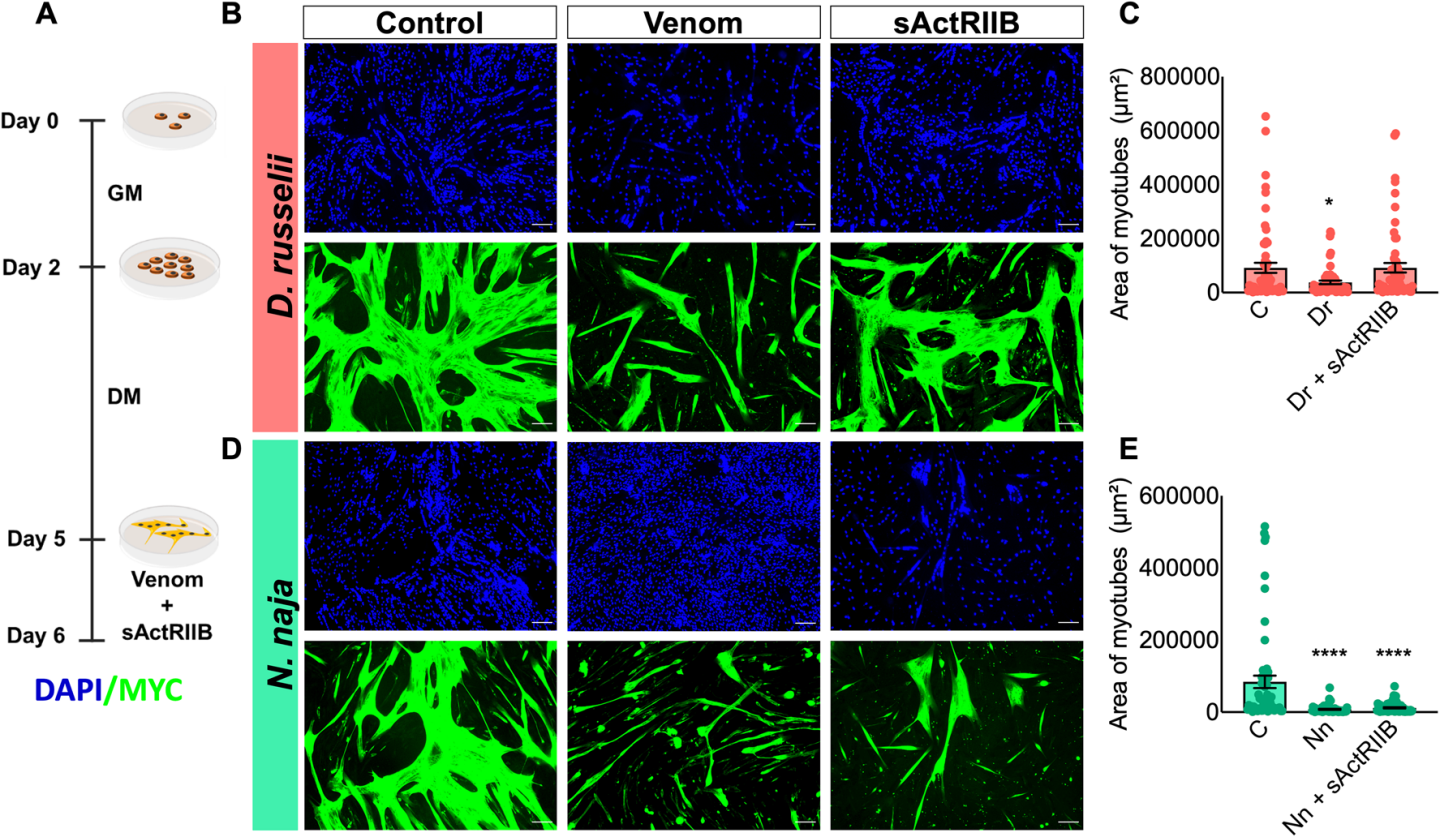
Effects of sActRIIB on venom-induced muscle atrophy. (**A**) Graphical representation of the atrophy experiment performed. Representative images of myotubes incubated with 1.25 µg/mL *D. russelii* (**B**) or *N. naja* (**D**) venom in the absence or presence of 1 µg/mL sActRIIB and quantification of the area of myotubes (**C**,**E**) are shown. As negative controls (**C**), myotubes were exposed to the differentiation medium alone. Data represent mean ± SEM (60 myotubes were assessed for each treatment). *P* values (*p < 0.05 and ****p < 0.0001) shown were as calculated by one-way ANOVA followed by Dunnett’s post hoc test using GraphPad Prism by comparing the treated groups with the negative controls. The scale bars represent 100 µm.

### sActRIIB prevents atrophy induced by *D. russelii* venom

Muscle atrophy is mediated by activin and myostatin, both of which affect the ability of Akt to support anabolic activity while activating catabolic programmes^25^. The sActRIIB is a potential pharmacological molecule for preventing muscle wasting through its ability to sequester activin and myostatin^26^. Therefore, sActRIIB was used as a ligand trap to bind activin/myostatin and allow muscle growth. Here, we assessed if this molecule will be effective in preventing venom-induced muscle atrophy. As shown in Fig. 6A, the myotubes were fully developed and treated with venoms and a specific concentration (1 µg/mL, as determined in previous studies) of sActRIIB. As expected, the control group exhibited typical myotube formation (Fig. 6B) and sActRIIB on its own slightly increased the area of myotubes (Figure S1). However, the morphology of differentiated cells incubated with venoms became irregular and the area was significantly decreased (Fig. 6B,D). The myotubes treated with *D. russelii* venom and sActRIIB were observed to have a similar myotube area compared to the controls (Fig. 6B,C). An increase in myotube area was observed in the sActRIIB and *D. russelii* venom-treated myotubes compared to those exposed to venom alone. However, sActRIIB did not show any effect in preventing *N. naja* venom-induced atrophy of myotubes (Fig. 6D,E). These data suggest that sActRIIB is an effective molecule to prevent *D. russelii* venom-induced atrophy although it did not exhibit any effect on *N. naja* venom-induced atrophy.

## Discussion

Skeletal muscle has a remarkable capacity to regenerate even after major damage. Therefore, it is intriguing that despite this property, it fails to regenerate following SBE by specific species including vipers. As early phases of muscle regeneration are crucial to repairing, in this study, we investigated which of these phases are affected by venoms of two clinically relevant snakes (Russell’s viper and Indian cobra). To the best of our knowledge, for the first time in this study, AB1190 myoblasts were utilised as an in vitro tool to evaluate the direct effects of *D. russelii* and *N. naja* venoms. The results from the MTS assay indicate that both venoms contain toxins that target cultured human myoblasts to modulate their viability. *D. russelii* venom mildly affected AB1190 cells, with the high concentrations decreasing the viability of myoblasts. Contrarily, *N. naja* induced a significant reduction in cell viability even at lower concentrations. Venoms from other snakes of the *Naja* genus, such as *Naja haje* and *Naja atra* have shown similar cytotoxic effects on murine myoblasts at comparable concentrations^27^. Therefore, the impaired muscle regeneration observed in humans following envenomation by *D. russelii* or *N. naja* could be due to a decrease in the number of mononucleated muscle cells that are required for tissue restoration.

Cell migration is a crucial phenomenon of muscle development and regeneration^28^. The cell migration assay revealed distinct patterns following exposure to *D. russelii* and *N*. *naja* venoms. The migration distance and speed declined when myoblasts were incubated with *D. russelii* venom. The migratory capacity of myoblasts is essential for wound healing, as myoblasts need to traverse the damaged monolayer before successful differentiation can occur. *D. russelii* venom significantly increased the time taken for cells to traverse and contact following the disruption of the myoblast monolayer. No significant effect was observed by any of the concentrations of *N. naja* venom investigated. The differential effects of these venoms on myoblasts in wound healing could also contribute to the delayed regeneration observed in viper bite victims and highlight a potential reason why delayed regeneration is not observed in *N. naja* envenomations. Some studies have highlighted the prevalence of disintegrins in viper venoms and their impact on inhibiting the migration of endothelial cells, neutrophils, and cancer cells by blocking selective integrin molecules on their surface^29^. Since *D. russelii* venom contains disintegrins and disintegrin-like proteins^30^, they may impact the migration of myoblasts. These proteins are rarely found in elapid venoms^31^ and therefore, these venoms may not adversely affect integrins-mediated cell migration. Previous studies have reported that an SVMP from *B. alternatus* reduces the migration of myoblasts in culture through mechanisms dependent and independent of its protease activity^32^. A biochemical study has reported that the proteome of *D. russelii* venom has diverse SVMP isoforms^33^. Thus, the participation of SVMPs in the inhibition of cell migration directly through cleaving integrins and indirectly by affecting the extracellular matrix cannot be ruled out. *N*. *naja* venom-treated cells did not show any alterations to cell migration at lower concentrations. However, 5 µg/mL *N. naja* venom slightly increased the migratory distance and speed although this effect was not observed when a scratch assay was performed. This warrants further investigation to establish if the *N. naja* venom indeed increases cell migration, and if so, establish the underlying mechanisms.

Since our data demonstrate that venoms modulate cell motility, the underlying mechanisms of these alterations were explored in this study by analysing focal adhesions and actin filaments, which are required to generate the tension necessary for traction. The size of focal adhesions is linked to a cell’s migratory ability^34^. Phalloidin and paxillin staining were used in our imaging analysis to quantify the amount of F-actin and focal adhesion scaffold in cells, respectively. In concordance with the migration data, *D. russelii* venom treatment for 24 h dramatically decreased the number and area of focal adhesions and F-actin levels. However, *N. naja* venom did not induce any changes to the number of focal adhesions or F-actin filaments. It appears that the focal adhesions might be a target for viper venom components, but further experiments are required to substantiate this notion. Cell migration can be increased by having a greater turnover of focal adhesions or through the assembly of less complex structures^35^. Furthermore, by critically analysing our data, we note that the changes in migration distance are much more of a sensitive parameter to the impact of venom than the assessment of the number or size of focal adhesions. These data suggest that other parameters including signalling mechanisms need to be considered to fully explain these findings. Overall, a deeper analysis of these differential effects of Russell’s viper and cobra venoms may form the basis for a better understanding of the differences in muscle degeneration/regeneration following SBE.

Myoblast fusion is one of the cornerstone processes during muscle regeneration^36^. Given its importance, the impact of venoms on the fusion of human myoblasts to form myotubes was analysed. Three days after the addition of differentiation media, mononucleated myoblasts incubated without venoms (control group) fused into multinucleated myotubes, generating the typical pattern of striated muscle. In contrast, *D. russelii* or *N*. *naja* venom reduced the areas of myotubes and the number of fused nuclei. Therefore, both venoms affected the process of fusion even at low concentrations. The fusion of myoblasts involves several processes, including recognition, adhesion, and membrane remodelling^37^. Many of the critical actors in these processes are surface proteins present in the myoblasts^38^. Hence, it is conceivable that venom proteolytic and/or PLA_2_ activity may affect these proteins and membrane integrity, respectively and thereby impair fusion. This impairment of cell fusion may contribute to attenuated regeneration following SBE. Incomplete myotube formation was also evidenced in C2C12 myoblasts following exposure to murine muscle homogenates obtained from envenomed mice by the venom of *Bothrops asper* (the most medically important pit viper in Central America)^39^. The long-term presence of toxins in muscle during different stages of regeneration has been detected in previous studies with viper venoms, and this impedes the complete regeneration of the muscle^40^. Atrophy assays showed that the control myotubes were larger than the ones treated with venoms. Interestingly, this effect was reported at concentrations lower than those inducing a reduction in myoblast viability. Thus, our comparative study sheds light on the differences in susceptibility of myoblasts and myotubes to these venoms. Myotubes were more sensitive to venoms than myoblasts, a pattern that was also previously highlighted by other studies^41^. Therefore, the concentrations of venoms used in this study differ based on the nature of the experiments performed. While the *D. russelii* venom contains a significant amount of SVMPs and PLA_2_^30,33^, *N. naja* venom has been shown to possess a large amount of PLA_2_ and 3FTXs^42^. Therefore, the impact of all these different toxins in inducing varying effects on myoblasts and thus their differentiation and atrophy cannot be ruled out.

Although several therapeutic options are being evaluated for SBE treatment, antivenoms are still the only validated treatment for this condition^43,44^. Usually, these life-saving medicines are not readily available in rural high-risk areas where incidences occur frequently. The time delay in receiving antivenom treatment following SBE often results in extensive local tissue damage that can lead to permanent disabilities^45,46^. As a result, there is an urgent need to develop better, more efficacious, and affordable treatments for SBE. A particular interest has been placed on broad-spectrum inhibitors that can inhibit specific venom toxins in a range of different venoms. Therefore, in recent years, the repurposing of small-molecule inhibitors for venom toxins has attracted toxicologists, medicinal chemists, and clinicians to determine their pre-clinical effects in various experimental settings^47^. Besides the promising effects, they are useful tools for identifying the biochemical composition of venoms and their mechanisms of toxicity. Given the myotoxic nature of SVMPs and PLA_2_s, we examined the ability of two small molecule inhibitors, prinomastat (an SVMP inhibitor) and varespladib (a PLA_2_ inhibitor) in this study to assess their ability to reverse the impact of the venoms on AB1190 myotubes. As expected, the antivenom that was used as a control attenuated the venom-induced myotube atrophy for both venoms. However, the inhibitors only had neutralising effects on *D. russelii* venom, not on *N. naja* venom. Our findings are consistent with the results of previous work demonstrating that varespladib aids in neutralising myotoxicity, but complete inhibition has not been observed^48^. This suggests that PLA_2_s and SVMPs play a key role in muscle atrophy induced by *D. russelii venom* and evidences the complexity of this venom and its mechanisms of toxicity. No protective effect was observed for *N. naja* venom with these two inhibitors indicating that PLA_2_s and SVMPs from this venom may not significantly contribute to its myotube toxicity. Other research groups have demonstrated that elapid venoms are rich sources of 3FTX family members with myotoxic effects^49^. Therefore, 3FTX-induced cytotoxic effects may play a critical role in *N. naja* venom-induced effects on the myoblasts. Thus, the neutralisation of 3FTXs and other non-enzymatic toxins must be evaluated for envenomation by elapid snakes. A multifaceted approach toward SBE treatment, combining inhibitors targeting catalytically active and medically important non-enzymatic toxins will result in more effective treatments for SBE. Since the antivenom rescued the effects induced by *N. naja* venom, 3FTX-specific antibodies might be useful to determine if these toxins are a critical player in *N. naja* venom-induced effects in myoblasts and myotubes.

The use of regenerative medicine approaches to promote muscle regeneration following acute and chronic muscle damage is being investigated widely to tackle various muscle disorders^50^. sActRIIB has been proposed as an attractive regenerative therapy for muscle-wasting diseases^51,52^. The benefits of this and other classes of activin/myostatin inhibitors arise from their ability to prevent activin/myostatin-mediated muscle loss^53–55^. Given the severe SBE-induced muscle damage characterised by significant atrophy, we hypothesised that sActRIIB could help prevent this process. Hence, we determined the effect of sActRIIB on AB1190 myotubes incubated with venoms. The use of sActRIIB attenuated the *D. russelii* venom-induced myotube atrophy. However, no protective effect from this treatment was observed in myotubes exposed to *N. naja* venom. These differences again highlight the variability in proteomic contents and distinct mechanisms of action of toxins in these two venoms. Based on these findings, we propose that *D. russelii* venom can increase the amount of biological activity of activin/myostatin, a process that is not stimulated by *N. naja* venom. On the other hand, *N. naja* venom might affect the myotubes independently from the activin/myostatin signalling axis. Activin/myostatin levels can be modulated by several processes at the levels of transcription and post-transcription, including their liberation from the ECM^56^. Therefore, further detailed studies are required to underpin the molecular mechanisms behind venom-induced muscle atrophy, and this will pave the way to develop better therapeutics for SBE-induced muscle damage.

## Conclusions

Our results using a range of experimental approaches in this study demonstrate that AB1190 human myoblasts are a promising in vitro tool for assessing the impacts of venoms on myogenic regulation, understanding SBE-induced muscle pathological conditions and screening emerging therapeutics for SBE. Moreover, assessing the various stages of muscle differentiation underpins the impact of venoms at distinct levels. The data demonstrate that *D. russelii* and *N. naja* venoms impact different phases of muscle development, but myotubes are more affected than myoblasts alone. Despite the similarities, *D. russelii* venom reduces the migration of myoblasts and the number of focal adhesions in contrast to *N. naja* venom. This major discriminating feature may be one of the reasons behind the poor muscle regeneration in victims following viper envenomation. Treatment of muscle atrophy-related diseases is challenging, but the venom variability further compounds this complexity. Varespladib and prinomastat as stand-alone therapies may not fully protect against viper-induced muscle atrophy and they do not mitigate *N. naja* venom-induced atrophy. Moreover, these data highlight the potential of sActRIIB as a treatment for SBE (at least for viper)-induced muscle damage. While several toxins in venoms may synergistically affect myoblasts and myotubes to induce muscle damage, SVMPs and PLA_2_s in *D. russelii* venom and 3FTXs in *N. naja* venom might be the major components to induce such effects. These data shed light on how the varying venom composition in these medically important snakes may affect SBE patients to induce diverse clinical complications relating to muscle damage. This also explains the severity of muscle damage induced by Russell’s viper and the rationale for this leading to permanent disabilities. This further emphasises the need to seek prompt hospital treatment to prevent excessive muscle damage and subsequent permanent disabilities in SBE victims. Together, this study provides a basis for future experimental research to determine the impact of combinational therapeutic approaches to ameliorate muscle damage induced by SBE.

### Supplementary Information

Supplementary Figure S1.

## Data Availability

The datasets used and/or analysed during the current study are available upon request from the corresponding author.
