# Supplementary material for: Differential effects of the venoms of Russell’s viper and Indian cobra on human myoblasts
Source: Sci Rep. 2024 Feb 7;14:3184. doi: 10.1038/s41598-024-53366-9 (PMC10850160; doi:10.1038/s41598-024-53366-9)
Supplement: Supplementary file 1 — Supplementary Figure S1. [file 41598_2024_53366_MOESM1_ESM.docx]

**Supplementary information**


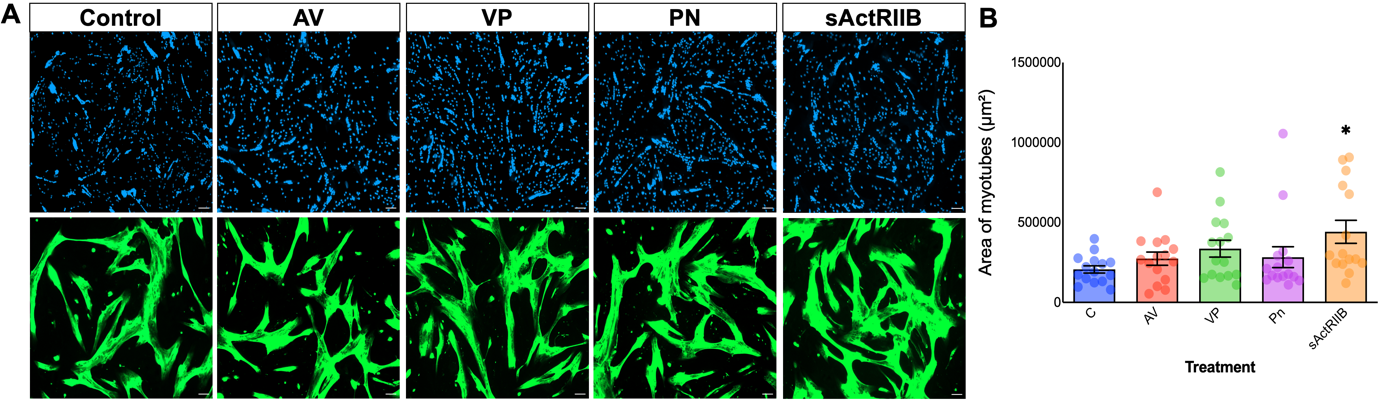


**Figure S1:** **Effects of small molecule inhibitors, antivenom and sActRIIB on differentiated myotubes in the absence of venoms.** (**A**) Representative images showing the control myotubes and the ones treated with the same concentrations (as used in the above experiments) of antivenom (AV), varespladib (VP), prinomastat (PN) and sActRIIB. **B**, the area of myotubes was quantified for all treated samples and compared with the control (C). Data represent mean ± SEM (15 myotubes were assessed for each treatment). P value (*p<0.05) shown was calculated by one-way ANOVA followed by Dunnett’s post hoc test using GraphPad Prism. The scale bars represent 100 µm.
